# A testing time for gradient strips: an evaluation of ETEST for the antimicrobial susceptibility testing of Neisseria gonorrhoeae

**DOI:** 10.1099/jmm.0.002088

**Published:** 2025-10-28

**Authors:** Rachel Pitt-Kendall, Jack Minshull, Sandhya Vivekanand, Sandra David, Helen Fifer, Michelle Cole, Sarah Alexander

**Affiliations:** 1 UK Health Security Agency, London NW9 5EQ, UK

**Keywords:** antimicrobial resistance, antimicrobial susceptibility testing, *Neisseria gonorrhoeae*

## Abstract

**Introduction.** Antimicrobial susceptibility testing (AST) of *Neisseria gonorrhoeae* isolates is recommended in the UK to ensure antimicrobial stewardship and detection of multi-drug and extensively resistant cases. In diagnostic and reference laboratories, this testing is primarily carried out via a gradient strip. However, agar dilution methodology may also be used for high-throughput testing.

**Gap statement.** *N. gonorrhoeae* is not a validated species on all ETEST (bioMérieux, France) gradient strip formulations available, and, therefore, additional comparative validation data are required to support use in clinical laboratory settings.

**Aim.** To determine the reproducibility of ETEST for AST of *N. gonorrhoeae*, and to demonstrate the comparability of susceptibility results obtained using agar dilution and gradient strip methods.

**Methodology.** Modal ETEST MICs for six well-characterized World Health Organization *N. gonorrhoeae* control strains, against eight antimicrobials, were calculated. ETEST modal MICs were compared to published and local, historical agar dilution MICs. MICs were interpreted using European Committee on Antimicrobial Susceptibility Testing (EUCAST) breakpoints. Concordance of ETEST modal MICs, within essential and categorical agreement, for each strain was calculated.

**Results.** Overall, 95.80% of modal ETEST MICs were within essential agreement with published MICs. Where variance from exact concordance was noted, a systematic shift was observed to lower MICs in this study. On three occasions, variance from the published MIC resulted in a categorical classification change. On two occasions, the modal ETEST MIC in this study was two doubling dilutions lower than the published MIC. Neither resulted in a categorical classification change. When modal ETEST and agar dilution MICs were compared, overall essential agreement was 83.30%. However, this increased to 94.4% when concordance was analysed for clinically important antimicrobials: azithromycin, cefixime and ceftriaxone. Again, a systematic shift to lower MICs for ETEST was observed in this study. Categorical agreement was 83.3% for all antimicrobials and 100% for clinically important antimicrobials.

**Conclusion.** Excellent concordance was demonstrated for MICs generated using ETEST with published MICs. Good concordance was observed for MICs generated using two different susceptibility testing methodologies. Where variance was noted, MICs generally read lower on ETEST in this study. ETEST remains fit for purpose for the AST of *N. gonorrhoeae*, a clinically important pathogen.

## Introduction

Antimicrobial resistance in *Neisseria gonorrhoeae* is a global public health concern, and consequently, it has been a World Health Organization (WHO) priority organism since 2012 [1]. As such, primary diagnostic laboratories in the UK are recommended to perform susceptibility testing of all confirmed *N. gonorrhoeae* isolates to ceftriaxone, the last-line antimicrobial for empirical treatment, as well as to other antimicrobials which may be used as alternative treatments [2]. This primarily enables early detection of ceftriaxone-resistant isolates, which are often multi-drug or extensively drug-resistant [3] and may require additional investigation, enhanced partner notification and follow-up to ensure successful treatment and prevention of onwards transmission.

Historically, many laboratories used the disc diffusion method for antimicrobial susceptibility testing (AST) of *N. gonorrhoeae* as it was inexpensive and efficient, allowing multiple antimicrobials to be tested at the same time on the same agar plate. However, there were notable issues with the correlation of zone diameters and the MICs for *N. gonorrhoeae* [4]. Furthermore, some specific antimicrobial discs, such as ceftriaxone, were not always available at testing laboratories, meaning that susceptibility was inferred from other members of the antibiotic class (e.g. cefuroxime). Consequently, the British Association for Antimicrobial Chemotherapy recommended the use of gradient strips in combination with EUCAST clinical breakpoints [5] for the susceptibility testing of this organism [4]. The previous BSAC methodology for disc diffusion testing was no longer supported.

The ETEST (bioMérieux, France) is one such brand of gradient strip system that comprises a plastic strip, impregnated with a pre-defined antimicrobial gradient. The strips allow determination of isolate MICs (mg l^−1^) after 18–24 h incubation, facilitating efficient reporting of clinical results. Previous evaluations of ETEST gradient strips have shown them to be reliable, producing comparable results to other gradient strips [6]. As such, this assay is commonly employed in the UK and internationally for antimicrobial susceptibility testing. However, a review of the ETEST Instruction For Use (IFU) identified that *N. gonorrhoeae* was not listed as a defined micro-organism for which testing had been validated, for azithromycin, cefixime, ertapenem or gentamicin. Additionally, it was recognized that *N. gonorrhoeae* was listed as a validated microorganism for benzyl-penicillin, ceftriaxone, ciprofloxacin and tetracycline, but not for all formulations of these (e.g. validated for low-concentration strips but not high-concentration strips). Consequently, clinical laboratories using ETESTs to determine the MICs for *N. gonorrhoeae* may be doing so outside of the scope of the manufacturers’ intended use, colloquially referred to as ‘off-label’. Further use of this *in vitro* medical diagnostic device for susceptibility testing of *N. gonorrhoeae* would, therefore, require validation data in order to comply with the Quality Management System requirements, which are in place for most clinical diagnostic laboratories [7].

The Sexually Transmitted Infections Reference Laboratory (STIRL) at the UK Health Security Agency (UKHSA) offers specialist reference services, surveillance activities and outbreak investigation for bacterial STIs, including *N. gonorrhoeae*. Gradient strip (namely ETEST) and agar dilution methods are used for the AST of *N. gonorrhoeae* isolates in STIRL.

The following report aims to provide (i) validation data that can be used to support clinical laboratories that use the ETEST system for AST for *N. gonorrhoeae* and (ii) to also understand the comparability of MICs generated by the agar dilution and gradient strip methods for this organism.

## Methods

### Control strain panel

Six well-characterized WHO control *N. gonorrhoeae* strains [WHO G (NCTC 13478), WHO K (NCTC 13479), WHO M (NCTC 13481), WHO O (NCTC 13483), WHO P (NCTC 13484) and WHO Q (NCTC 14208)] [8,9], representing varied antibiograms, were selected for this evaluation.

### ETEST evaluation

To assess reproducibility, ETESTs (bioMérieux, France) were performed in triplicate for each WHO control strain and antimicrobial tested as per the manufacturer’s instructions. ETESTs were evaluated for the following antimicrobials: azithromycin (catalogue number: 412257, 0.016–256 µg ml^−1^), benzyl-penicillin (catalogue number: 412265, 0.002–32 µg ml^−1^), cefixime (catalogue number: 412275, 0.016–256 µg ml^−1^), ceftriaxone (catalogue number: 412301, 0.016–256 µg ml^−1^), ciprofloxacin (catalogue number: 412311, 0.002–32 µg ml^−1^), ertapenem (catalogue number: 412332, 0.002–32 µg ml^−1^), gentamicin (catalogue number: 4122368, 0.016–256 µg ml^−1^) and tetracycline (catalogue number: 412471, 0.016–256 µg ml^−1^). Briefly, pure 18–24 h cultures on non-selective, GCVIT media (GC agar base, BD Difco plus 1% Vitox, Oxoid) were used to make 0.5 McFarland suspensions in sterile saline. Strains were inoculated onto GCVIT plates and ETESTs applied. Plates were incubated at 37 °C, 5% CO_2_ for 24 h, after which the MICs were assigned where the ellipse of growth inhibition intersected the strip. If the ellipse intersected between two dilutions, the MIC was read at the higher dilution. All actual MICs were converted to the doubling dilution (Log_2_) scale for analysis. MICs were interpreted using EUCAST breakpoints where available [5].

For each strain and antimicrobial combination, modal MICs were calculated and compared to published MIC data for these strains [8,9]. The MICs in these publications are used by laboratories globally as the expected values for these control strains. Therefore, comparison of data generated in this study with these expected values indicated assay reproducibility. Variance of MICs was analysed with respect to the initial triplicate (reproducibility) and then also in comparison to the published data. MICs that were ≤one doubling dilution different from each other were within essential agreement. This varies from the standard definition where MICs are compared to the reference method, not to each other, to establish essential agreement [10]. Where MICs differed by two or more doubling dilutions, these were considered outside of essential agreement and a fault.

### Comparison with agar dilution

The modal MICs generated for each strain and antimicrobial combination using ETEST were compared with longitudinal modal MICs generated by agar dilution through the Gonococcal Resistance to Antimicrobials Surveillance Programme (GRASP) on GCVIT media [11,12]. Full MICs are established for azithromycin (range 0.06–4 mg l^−1^), cefixime (range 0.004–0.5 mg l^−1^), ceftriaxone (range 0.004–0.25 mg l^−1^) and tetracycline (range 0.25–8 mg l^−1^). A breakpoint plate is used for benzyl-penicillin at 1 mg l^−1^. MICs are interpreted using EUCAST breakpoints [5]. A panel of well-characterized WHO control strains (those described above) is used as internal quality controls for each run of susceptibility testing. Twenty-two data points, generated between November 2024 and April 2025, were available for each quality control strain for comparison in this analysis. These were used to calculate the reference modal MICs for the agar dilution method in this analysis. For agar dilution susceptibility testing runs to be valid, control strain MICs must have been within one doubling dilution of the expected MICs [8,9].

Agar dilution MICs were prepared as follows. Isolate suspensions (0.5 McFarland standard) in sterile saline were spotted using a multi-point inoculator onto GCVIT agar containing serial dilutions of selected antimicrobials. Plates were incubated for 24 h at 37 °C, 5% CO_2_, prior to reading. MICs were assigned to the lowest concentration of each antimicrobial that inhibited growth.

MICs were compared for the following antimicrobials: azithromycin, benzyl-penicillin, cefixime, ceftriaxone and tetracycline. Phenotypic susceptibility testing is no longer carried out as part of GRASP for other antimicrobials. As GRASP tests antimicrobials that are historical as well as currently recommended treatment options, further analysis on concordance of results was performed only for clinically important antimicrobials (azithromycin, ceftriaxone and cefixime).

## Results

### ETEST reproducibility

When the strain panel was tested in triplicate, 93.8% (135/144) of MICs were the same as, and 100% were within one doubling dilution of the modal MIC for each strain and antimicrobial combination. Where variance was noted, none of the differences changed the susceptibility category assigned when EUCAST breakpoints [5] were applied (where appropriate).

### Comparison with published MICs

The modal ETEST MICs were compared to the published MICs for these strains (Table 1) [8,9]. For 52.1% (25/48) MICs, the ETEST modal MIC and published MIC were the same. A further 43.8%(21/48) MICs were within one doubling dilution of each other and, therefore, within essential agreement. Of the MICs that read one doubling dilution different, 95.2% (20/21) were read lower on ETEST than the published MIC that was also established using ETEST. For three strains and antimicrobial combinations, the one doubling dilution variance resulted in a change to the susceptibility category reported. These were WHO K and benzyl-penicillin, WHO G and ciprofloxacin and WHO P and tetracycline (highlighted in bold on Table 1). In all cases, the published MIC was reported as resistant whilst the ETEST modal MIC was reported as susceptible.

**Table 1.** Comparison of modal MICs generated using ETEST with the published [8,9] MICs, for six well-characterized *N. gonorrhoeae* control strains

			WHO G		WHO K		WHO M		WHO O		WHO P		WHO Q	
	EUCAST breakpoint (mg l^−1^) [5]		MIC (mg l^−1^)	S/R	MIC (mg l^−1^)	S/R	MIC (mg l^−1^)	S/R	MIC (mg l^−1^)	S/R	MIC (mg l^−1^)	S/R	MIC (mg l^−1^)	S/R
Azithromycin	ECOFF 1	Modal	0.125	n/a	0.125	n/a	0.25	n/a	0.25	n/a	2	n/a	>256	n/a
		Published	0.25		0.5		0.5		0.5		4		>256	
Benzyl-penicillin	>1	Modal	0.5	S	**1**	**S**	>32	R	>32	R	0.125	S	1	S
		Published	0.5	S	**2**	**R**	>32	R	>32	R	0.25	S	1	S
Cefixime	>0.125	Modal	≤0.016	S	0.25	R	≤0.016	S	≤0.016	S	≤0.016	S	1	R
		Published	<0.016	S	0.25	R	≤0.016	S	0.016	S	≤0.016	S	2	R
Ceftriaxone	>0.125	Modal	≤0.016	S	0.125	S	≤0.016	S	0.016	S	≤0.016	S	0.5	R
		Published	0.008	S	0.064	S	0.016	S	0.03	S	0.004	S	0.5	R
Ciprofloxacin	>0.06	Modal	**0.064**	**S**	>32	R	1	R	0.008	S	0.004	S	>32	R
		Published	**0.125**	**R**	>32	R	2	R	0.008	S	0.004	S	>32	R
Ertapenem	n/a	Modal	0.008	n/a	0.064	n/a	0.004	n/a	0.008	n/a	0.004	n/a	0.016	n/a
		Published	0.008		0.064		0.016		0.016		0.004		0.032	
Gentamicin	n/a	Modal	2	n/a	2	n/a	4	n/a	4	n/a	4	n/a	2	n/a
		Published	4		4		4		4		4		4	
Tetracycline	>0.5	Modal	16	R	1	R	2	R	1	R	**0.5**	**S**	32	R
		Published	32	R	2	R	2	R	2	R	**1**	**R**	128	R

ECOFF, epidemiological cut-off; R, resistant; S, susceptible.

There were two (4.2%) instances where the ETEST modal MIC was two doubling dilutions different from the published MIC, both lower. This was for WHO K and azithromycin (ETEST modal MIC: 0.125 mg l^−1^ vs. published MIC: 0.5 mg l^−1^ [9]) and for WHO Q and tetracycline (ETEST modal MIC: 32 mg l^−1^ vs. published MIC: 128 mg l^−1^) (Table 1). These would be categorized as faults. Where EUCAST breakpoints were applied, neither resulted in a move across an epidemiological cut-off breakpoint or a change to the susceptibility category assigned [5]. It should be noted that the published WHO K MIC for azithromycin was reported as 0.25 mg l^−1^ in the original WHO panel [8]. This was superseded by the publication of the updated panel data and is now 0.5 mg l^−1^ [9]. If the reference MIC from the 2016 publication was used as the comparator in this analysis, the azithromycin ETEST MIC for this strain would be within essential agreement.

### Comparison with agar dilution

The modal MICs generated by ETEST were compared with the modal MICs generated by GRASP for these strains using the agar dilution method and the published MICs (Table 2). The use of a breakpoint plate in GRASP for benzyl-penicillin meant that it was not possible to directly compare the results for this antimicrobial. However, it was possible to comment on the degree of concordance of the susceptibility category.

**Table 2.** Comparison of modal MICs generated using ETEST with modal MICs generated by agar dilution and published MICs [8,9], for six well-characterized *N. gonorrhoeae* control strains

			WHO G		WHO K		WHO M		WHO O		WHO P		WHO Q	
	EUCAST breakpoint (mg l^−1^)		MIC (mg l^−1^)	S/R	MIC (mg l^−1^)	S/R	MIC (mg l^−1^)	S/R	MIC (mg l^−1^)	S/R	MIC (mg l^−1^)	S/R	MIC (mg l^−1^)	S/R
Azithromycin	ECOFF 1	ETEST modal	0.125	n/a	0.125	n/a	0.25	n/a	0.25	n/a	2	n/a	>256	n/a
		Agar dilution	0.25		0.5		0.5		0.5		4		>4	
		Published	0.25		0.5		0.5		0.5		4		>256	
Benzyl-penicillin	>1	ETEST Modal	0.5	S	**1**	**S**	>32	R	>32	R	0.125	S	1	S
		Agar dilution	≤1	S	**>1**	**R**	>1	R	>1	R	≤1	S	>1	S
		Published	0.5	S	**2**	**R**	>32	R	>32	R	0.25	S	1	S
Cefixime	>0.125	ETEST modal	≤0.016	S	0.25	R	≤0.016	S	≤0.016	S	≤0.016	S	1	R
		Agar dilution	0.032	S	0.5	R	0.016	S	0.032	S	0.032	S	>0.5	R
		Published	<0.016	S	0.25	R	≤0.016	S	0.016	S	≤0.016	S	2	R
Ceftriaxone	>0.125	ETEST modal	≤0.016	S	0.125	S	≤0.016	S	0.016	S	≤0.016	S	0.5	R
		Agar dilution	0.016	S	0.125	S	0.016	S	0.032	S	0.016	S	>0.25	R
		Published	0.008	S	0.064	S	0.016	S	0.03	S	0.004	S	0.5	R
Tetracycline	>0.5	ETEST modal	16	R	1	R	2	R	1	R	**0.5**	**S**	32	R
		Agar dilution	>8	R	4	R	4	R	4	R	**2**	**R**	>8	R
		Published	32	R	2	R	2	R	2	R	**1**	**R**	128	R

N.B. A benzyl-penicillin breakpoint plate was used to categorize isolates as susceptible or resistant.

ECOFF, epidemiological cut-off; R, resistant; S, susceptible.

Where it was possible to compare the MICs, 41.7% (10/24) MICs were the same for both AST methodologies (ETEST and agar dilution), whilst a further 41.7 %(10/24) were within one doubling dilution and essentially agreed (Table 2). However, there was a systematic shift to lower MICs when ETEST was used in comparison to agar dilution. All MICs within essential agreement were lower on ETEST than agar dilution. This shift was confirmed when the agar dilution MICs were compared to the published MICs, which were also generated using ETEST [8,9]. In this comparison, 95.8% (23/24) MICs were within essential agreement, and where variance was noted (10/24, 41.7%), all published MICs were lower in comparison to the agar dilution MICs (Table 2). On 4/24 (16.7 %) occasions, the MICs using ETEST were two doubling dilutions different, all lower than the MICs on agar dilution. Additionally, there was 1/24 (4.2 %) occasion where the ceftriaxone published MIC was two doubling dilutions lower than the agar dilution MIC (WHO P, agar dilution MIC: 0.016 mg l^−1^ vs published MIC: 0.004 mg l^−1^).

As mentioned previously, benzyl-penicillin is tested using a breakpoint plate in GRASP, which allows differentiation of susceptible and resistant isolates without giving an actual MIC. When the two methodologies were compared, the category of susceptibility was concordant for 5/6 (83.3 %) of the WHO strains tested (Table 2). With the only discordant result being WHO K (highlighted in bold, Table 2).

There was one further occasion where an MIC variance between the two testing methodologies resulted in a change to the susceptibility category reported. This was for WHO P and tetracycline, where the ETEST modal MIC (0.5 mg l^−1^) was reported as susceptible and the agar dilution MIC (2 mg l^−1^) was reported as resistant (highlighted in bold on Table 2).

### Overall essential agreement

Essential agreement of the ETEST gradient system in comparison to the published MICs was >95% (Table 3). According to Clinical & Laboratory Standards Institute (CLSI) guidance, this indicates that this method produces reliably reproducible results [13]. Overall essential agreement between the two different testing methodologies (ETEST and agar dilution) was lower at 83.3% (Table 3). Using CLSI-defined criteria, this level of concordance indicates that MICs are less reliably reproducible between the two different susceptibility testing methods [13]. However, when restricting to clinically important antimicrobials only (i.e. azithromycin, cefixime and ceftriaxone specifically), the degree of concordance between the ETEST and agar dilution MICs increases to 94.4% (Table 3).

**Table 3.** Summary of overall concordance of MICs results obtained via ETEST, in comparison with published MICs [8,9] for the strains tested and with agar dilution modal MICs

	MICs		Essential agreement*	Categorization†	
	Same	One doubling dilution		Same	Different
ETEST reproducibility	135/144 (93.8 %)	9/144 (6.3%)	100%	n/a	
ETEST vs. published MICs	25/48 (52.1%)	21/48 (43.8%)	95.80%	24/30 (80 %)	6/30 (20 %)
ETEST vs. agar dilution	10/24 (41.7%)	10/24 (41.7 %)	83.30%	20/24 (83.3 %)	4/24 (16.7 %)
ETEST vs agar dilution-CI‡	8/18 (44.4 %)	9/18 (50%)	94.4%	12/12 (100 %)	0/12

*MICs within one doubling dilution of the modal MIC, †susceptible or resistant, ‡CI – clinically important antimicrobials only: azithromycin, cefixime and ceftriaxone.

Categorical agreement was similar across the different susceptibility testing methodologies and was most commonly affected by MICs at or around the EUCAST breakpoint (Tables2  3) [5]. There was 100% categorical concordance between ETEST and agar dilution MICs generated for clinically important antimicrobials (Table 3).

## Discussion

Excellent reproducibility and concordance of MICs were generated for *N. gonorrhoeae* control strain isolates, using the ETEST gradient strip system. For all antibiotics tested, essential agreement was 100% for replicates within the same strain, similar to that reported previously [14,16]. Additionally, inter-lab concordance of MICs (within essential agreement) was >95 % when modal MICs were compared to published MICs for these strains [8,9] (Table 3). This is perhaps unsurprising given that the published MICs were generated using the same method that was as in this study (e.g. strains tested in triplicate to generate modal MICs), albeit using ETEST gradient strips on a different media [e.g. base (GC agar), BD Difco plus 1% haemoglobin, BD and 1% isovitalex, BD] [8,9].

Despite evidence of high levels of agreement of MICs generated using ETEST, there were some systematic differences. This was of particular note for azithromycin and tetracycline, where 83.3% (10/12, Table 1) of modal MICs were at least one doubling dilution lower than the published MICs for the strains tested. This resulted in a shift across an EUCAST breakpoint [5] on one occasion (WHO P, tetracycline), where the published MIC was reported as resistant, but the modal MIC in this study was reported as susceptible. There were two further occasions where MIC differences changed the susceptibility category reported (WHO G, ciprofloxacin and WHO K, benzyl-penicillin, Table 1). On both occasions, the published MIC for the strains was on the EUCAST breakpoint [5], and the reported MIC from this study was within one doubling dilution and therefore accepted variance.

Previous studies have shown that the performance of both azithromycin and tetracycline *in vitro* is affected by the pH of the media during incubation. Even small shifts in pH (e.g. pH 7.2 to 6.6) can reduce the potency of azithromycin [17,18]. Conversely, media pH levels <7.2 may increase the *in vitro* activity of tetracycline [18]. Surprisingly, if media pH levels exceed 7.4, the suppression of azithromycin and amplification of tetracycline activity may be reversed [18]. It could be that the differences seen in the MICs between this study and the published MICs are a result of a media component difference, e.g. the 1% haemoglobin, to some extent. However, as the antimicrobials require differing changes to pH to increase activity, and we did not observe an increase in tetracycline activity, media component differences do not fully explain the systematic shifts observed. Slight batch-to-batch variation of the antimicrobial concentrations contained within the gradient strips may also account for differences observed. However, the high level of MIC concordance with the published MICs for these strains, which were tested over a number of different time periods, does indicate the continued quality of the reagents.

The current gold standard method for susceptibility testing of *N. gonorrhoeae* is agar dilution. However, this method is time-consuming and labour-intensive, so it has limited application outside of surveillance programmes. At UKHSA, the agar dilution method is used in GRASP and ETEST to confirm MICs greater than the agar dilution range tested. As such, a comparison was performed of the comparability of MICs generated using the two different testing methodologies to give confidence in the concordance and accuracy of results. Due to differences in the antimicrobials tested through GRASP, modal MIC data were available for five antimicrobials only (Table 2). Where comparison of modal MICs was possible, 83.3% of MICs were within essential agreement (Table 3). However, concordance of the susceptibility category reported was higher, at 91.7% (44/48) (Table 2). When this analysis was restricted to clinically important antimicrobials (i.e. azithromycin, cefixime and ceftriaxone specifically), essential agreement of MICs generated using the two methodologies increased to 94.4% (Table 3), just below the CLSI threshold of ≥95% concordance to indicate reliably reproducible results [13]. Categorical concordance was 100%. These data give greater confidence in the accuracy of susceptibility testing processes; however, further evaluation of clinical strains would enhance this. Previous studies comparing clinical MICs generated using ETEST with agar dilution found that essential agreement (within one doubling dilution) ranged from 67.0 to 99.0 % and categorical concordance ranged from 85.0 to 100 %, depending on the antimicrobial tested and the growth medium used [14,16,19]. The lowest concordance was generally seen for the cephalosporins, in particular cefixime, in some studies [15,19, 20]. It is likely that more variability was seen with the clinical strains due to the more diverse range of MICs being tested in comparison to the control strain panel tested here.

Similarly to the comparison of modal MICs to the published data, systematic shifts to lower MICs were observed for those generated by ETEST for azithromycin, cefixime and tetracycline in comparison to agar dilution. This is in line with data previously reported in some studies, where ETEST generated MICs that were lower than agar dilution for some antimicrobials [15,19, 21]. Interestingly, a number of studies found the converse to be true, that MICs were higher on ETEST than agar dilution. This was noted for azithromycin and ceftriaxone [16,20]. Again, media differences between the studies likely contribute to this.

Whilst for most of the variances noted in this study, a one doubling dilution difference was observed, for 16.7% (4/24) of variances, ETEST MICs were two doubling dilutions different from the agar dilution modal MIC (Table 2). These would be considered faults. On three occasions, the two doubling dilution differences were seen when tetracycline was tested (i.e. WHO K, WHO O and WHO P) (Table 2). Despite this, the variance resulted in a change of susceptibility category on only one occasion (WHO P), where the agar dilution modal MIC was reported as resistant and the ETEST modal MIC reported as susceptible.

All cefixime modal ETEST MICs were within essential agreement of the modal agar dilution MICs. The modal cefixime ETEST MICs reported were consistently lower than the agar dilution modal MICs for WHO strains that were very susceptible (MIC ≤0.032 mg l^−1^, 4 of 6 WHO strains tested) (Table 2). For all four strains, the modal ETEST MIC was ≤0.016 mg l^−1^ and, therefore, beyond the lower limit of the antibiotic gradient on the strip. At very low concentrations of antimicrobial, whilst the MIC precision is less accurate, the impact is lower, as clinically this is irrelevant for patient management.

Phenotypic antimicrobial susceptibility testing is reliant on biological processes that may be affected by a range of factors. For example, the age of the culture used for inoculum preparation, the standardization of the inoculum concentration, the agar base used and any growth supplements added, the fastidiousness of the organism being tested, the quality and consistency of the reagents used and the method used to perform the testing may all affect the MIC obtained. Furthermore, interpretation of MICs is subjective and is vulnerable to operator inconsistencies, and there may be some slight differences between gradient strip batches. These factors, in addition to media pH, likely account for the differences seen in this report. The limitations of susceptibility testing have long been recognized by the scientific community, and as a result, the accepted tolerance of reproducibility within one doubling dilution (i.e. essential agreement) is applied globally.

This analysis has some recognized limitations. The MICs analysed for ETEST and agar dilution were not generated at the same time, from the same suspensions. So, whilst general trends in MICs can be observed, these results are not directly comparable. Additionally, it was not possible to perform bias calculations as part of this analysis as cell numbers per antimicrobial were <25 [10]. As such, we were unable to comment on whether the MIC variances seen using the ETEST method were a result of a systematic error, e.g. poor quality reagents which would affect all results, or as part of the natural variation of MICs within the tolerable limits due to reasons such as those described previously. Finally, it should be noted that the published MICs, used as a comparator in this study, were generated by a single testing laboratory. Generation of global modal MIC data for these strains would allow greater confidence in the expected value and in the concordance of results in studies such as this.

To conclude, we have demonstrated excellent reproducibility of MICs for *N. gonorrhoeae* using ETEST. We have also demonstrated excellent inter-laboratory concordance of modal MICs generated using ETEST, and through this, the continued performance of the ETEST gradient strip system since the generation of the initial published MICs for these control strains. Finally, we have demonstrated good concordance of modal MICs generated using two different antimicrobial susceptibility testing methods and very good concordance of susceptibility category reported using these methods. Overall, the data presented here support the continued use of ETEST for the antimicrobial susceptibility testing of *N. gonorrhoeae*. Continued susceptibility testing of this organism is imperative to retain it as a treatable infection.

## Abbreviations

AST: antimicrobial susceptibility testing
CLSI: Clinical & Laboratory Standards Institute
EUCAST: European Committee on Antimicrobial Susceptibility Testing
GRASP: Gonococcal Resistance to Antimicrobials Surveillance Programme
STIRL: Sexually Transmitted Infections Reference Laboratory
UKHSA: UK Health Security Agency
WHO: World Health Organization
